# Assessment of electrical dyssynchrony in cardiac resynchronization therapy: 12-lead electrocardiogram vs. 96-lead body surface map

**DOI:** 10.1093/europace/euac159

**Published:** 2022-09-15

**Authors:** Ksenia A Sedova, Peter M van Dam, Agnese Sbrollini, Laura Burattini, Lucie Necasova, Marie Blahova, Jan Bocek, Marek Sramko, Josef Kautzner

**Affiliations:** Department of Biomedical Technology, Faculty of Biomedical Engineering, Czech Technical University in Prague, Sitna Sq. 3105, 27201 Kladno, Czech Republic; Department of Cardiology, University Medical Center Utrecht, Heidelberglaan 100, 3584 CX Utrecht, The Netherlands; Department of Information Engineering, Università Politecnica delle Marche, via Brecce Bianche 12, 60131 Ancona, Italy; Department of Information Engineering, Università Politecnica delle Marche, via Brecce Bianche 12, 60131 Ancona, Italy; Department of Cardiology, Institute for Clinical and Experimental Medicine, Vídeňská 1958/9, 140 21 Prague 4, Czech Republic; Department of Cardiology, Institute for Clinical and Experimental Medicine, Vídeňská 1958/9, 140 21 Prague 4, Czech Republic; Department of Cardiology, Institute for Clinical and Experimental Medicine, Vídeňská 1958/9, 140 21 Prague 4, Czech Republic; Department of Cardiology, Institute for Clinical and Experimental Medicine, Vídeňská 1958/9, 140 21 Prague 4, Czech Republic; Department of Cardiology, Institute for Clinical and Experimental Medicine, Vídeňská 1958/9, 140 21 Prague 4, Czech Republic

**Keywords:** Body surface potential mapping, ECG imaging, Heart failure, Cardiac resynchronization therapy, LV lead positioning, AV delay optimization

## Abstract

**Aims:**

The standard deviation of activation time (SDAT) derived from body surface maps (BSMs) has been proposed as an optimal measure of electrical dyssynchrony in patients with cardiac resynchronization therapy (CRT). The goal of this study was two-fold: (i) to compare the values of SDAT in individual CRT patients with reconstructed myocardial metrics of depolarization heterogeneity using an inverse solution algorithm and (ii) to compare SDAT calculated from 96-lead BSM with a clinically easily applicable 12-lead electrocardiogram (ECG).

**Methods and results:**

Cardiac resynchronization therapy patients with sinus rhythm and left bundle branch block at baseline (*n* = 19, 58% males, age 60 ± 11 years, New York Heart Association Classes II and III, QRS 167 ± 16) were studied using a 96-lead BSM. The activation time (AT) was automatically detected for each ECG lead, and SDAT was calculated using either 96 leads or standard 12 leads. Standard deviation of activation time was assessed in sinus rhythm and during six different pacing modes, including atrial pacing, sequential left or right ventricular, and biventricular pacing. Changes in SDAT calculated both from BSM and from 12-lead ECG corresponded to changes in reconstructed myocardial ATs. A high degree of reliability was found between SDAT values obtained from 12-lead ECG and BSM for different pacing modes, and the intraclass correlation coefficient varied between 0.78 and 0.96 (*P* < 0.001).

**Conclusion:**

Standard deviation of activation time measurement from BSM correlated with reconstructed myocardial ATs, supporting its utility in the assessment of electrical dyssynchrony in CRT. Importantly, 12-lead ECG provided similar information as BSM. Further prospective studies are necessary to verify the clinical utility of SDAT from 12-lead ECG in larger patient cohorts, including those with ischaemic cardiomyopathy.

## Graphical Abstract

**Graphical Abstract:**
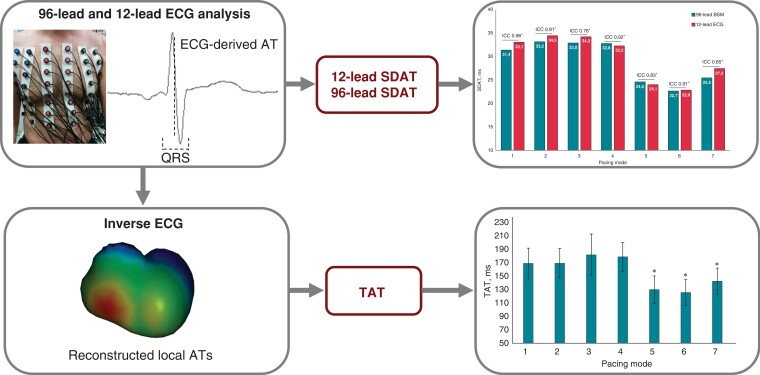

**What’s new?**

- The standard deviation of activation time (SDAT) derived from body surface mapping (BSM) is considered a reliable tool for assessing electrical dyssynchrony.
- This was confirmed in patients with non-ischaemic cardiomyopathy and cardiac resynchronization therapy (CRT) by the observation that changes in SDAT calculated from BSM corresponded to changes in reconstructed myocardial metrics of depolarization heterogeneity (total activation time, SDATm).
- Importantly, we demonstrated that the measurement of SDAT using 12-lead electrocardiogram (ECG) provides similar results as 96-lead BSM.
- The lowest SDAT values corresponded to the narrowest QRS duration during CRT with a pacing configuration that enables fusion with normal conduction (i.e. AV delay 20 ms shorter than spontaneous PQ interval).
- This observation warrants further clinical studies on the utility of 12-lead ECG for SDAT measurement.

## Introduction

Cardiac resynchronization therapy (CRT) is a recommended non-pharmacological therapy for patients with heart failure (HF) and reduced ejection fraction (HFrEF) and intraventricular conduction abnormalities. It provides both symptomatic relief and survival benefit and is associated with fewer hospitalizations for HF.^1,2^ However, not all patients respond favourably to CRT. Besides clinical parameters such as aetiology, HF, or gender, QRS duration and morphology is another predictor of the outcome and is used as an inclusion criterion in all available clinical trials. Given that the primary goal of CRT is to restore electrical synchrony through optimally timed biventricular pacing, quantifying electrical dyssynchrony appears to be essential for the prediction of CRT outcomes and optimizing the pacing settings.

The clinically used method to measure electrical dyssynchrony is QRS duration derived from standard 12-lead electrocardiogram (ECG), but the correlation of QRS duration with the response to CRT is generally not very high.^3^ More recently, body surface mapping (BSM) or the ECG imaging (ECGi) approach was proposed as a better alternative. In this respect, different indices for assessing depolarization heterogeneity have been suggested to improve CRT programming and LV lead placement.^4^ Among them, the standard deviation of activation time (SDAT) calculated from BSM is one of the most prospective indices of electrical dyssynchrony. Recent studies showed that the SDAT might help guide the CRT LV lead placement and device optimization.^5,6^ Some studies have explored the feasibility of the limited array of leads, using a special belt with 40 leads.^7^ Interestingly, no data comparing the directly diagnostic yield of 12-lead ECG compared with BSM are available. Therefore, the goal of this study was two-fold: (i) to compare the values of SDAT in individual CRT patients with reconstructed myocardial metrics of depolarization heterogeneity using an inverse solution algorithm and (ii) to compare SDAT calculated from 96-lead BSM with a clinically easily applicable 12-lead ECG.

## Methods

### Study population

We studied 19 patients with non-ischaemic dilated cardiomyopathy and HFrEF. They all had CRT systems implanted at least 6 months before BSM measurements. All patients were in sinus rhythm with QRS duration ≥120 ms and New York Heart Association (NYHA) Classes II and III HF, and were on optimal medical therapy prior to CRT for at least 3 months. The local ethics committee approved this study protocol and all patients gave informed consent. Patients were evaluated during a regular outpatient visit.

### Body surface map and 12-lead electrocardiogram

**Figure 1:**
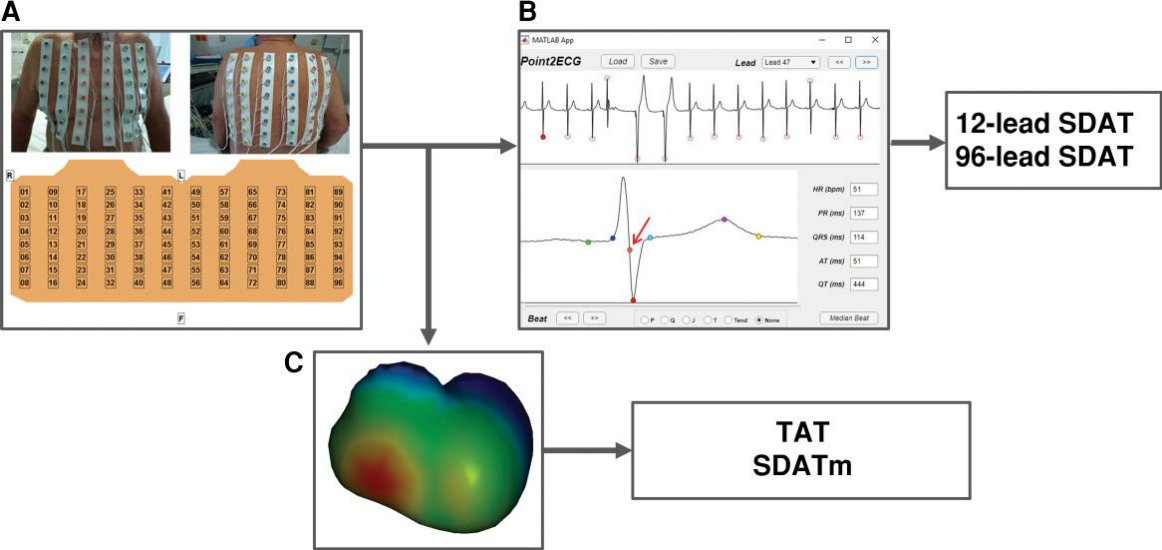
Body surface mapping (*A*) for measurement of 96- and 12-lead SDAT, indicated by an arrow (*B*) and inverse reconstruction of myocardial activation times to determine TAT and SDATm (*C*). SDAT, standard deviation of activation time; SDATm, standard deviation of myocardial activation time; TAT, total activation time.

Multichannel ECG signals were recorded from standard limb leads and unipolar 96 chest leads (interelectrode distance 3–5 cm) using a computer mapping system ProCardio-8 (16 bits; bandwidth 0.05–200 Hz; sampling frequency 1 kHz).^8^ Front and back thorax ECG electrodes were organized in 12 strips with 8 leads consistently (*Figure 1*). Six chest leads composed precordial leads and were used for the analysis of standard 12-lead ECG.

All ECG signals were prefiltered with a digital bandpass bidirectional Butterworth filter with cut-off frequencies of 0.5 and 40 Hz. R-peak positions were computed by Pan–Tompkins algorithm^9^ on Limb Lead II. Each lead was segmented into *N* (*N* being the number of beats in the recordings) ECG complexes, defined as segments between 350 and 450 ms before and after each R-peak position, respectively. Then, in order to remove possible ectopic beats, all ECG beats were compared among each other, computing the Pearsons’ correlation coefficient; only ECG complexes with a Pearsons’ correlation coefficient >0.85 were selected and executed in order to obtain the median ECG beat.

The median ECG beat was processed in order to compute the QRS onset and the QRS end automatically.^10^ The activation time (AT) point was defined as the minimum of the first-time derivative of potential during the QRS complex. Electrocardiogram signals with inappropriate quality due to bad contact with skin were discarded.

Thus, AT was automatically determined for each ECG lead in reference to the earliest AT in the set of 96 or 12 leads. Calculation of SDAT was performed automatically using either 96 thorax leads or standard 12 leads. QRS duration was measured manually in each of three standard limb leads by an independent observer who was not familiar with other measurements or the patient’s status. The averaged value of QRS duration was used for the analysis.

### Computed tomography examination

All patients underwent a non-contrast computed tomography scan of the chest after BSM recordings with the array of BSM electrodes *in situ* (SOMATOM Flash, dual-source scanner, 128-slice; Siemens Healthineers, Erlangen, Germany). The resulting images were used subsequently for the reconstruction of patient-specific models, incorporating thorax, myocardial surface, blood cavities, and the 96 electrode positions of BSM.

### Reconstructed myocardial activation times

To compare the SDAT values with the calculated myocardial activation sequences in each patient [i.e. total AT (TAT)], the latter parameter was non-invasively determined using the method of ECGi described by Boonstra *et al*.^11^ For this purpose, the above patient-specific models of the chest with electrode positions were employed. The estimation procedure to localize the activation sequence is a two-step process. In the first step, the stimulation site is roughly determined by the 3D direction of the QRS axis. In the subsequent step, the option of fusion activation with the intrinsically activated His-Purkinje system is added to the initial estimate.^11^ Both the foci positions and timing are optimized in a subsequent iterative procedure, such that the correlation between simulated and measured ECG signals is optimal. The resulted myocardial sequence of ventricular depolarization was used to obtain the myocardial parameters of the ventricular depolarization heterogeneity. Total AT was calculated as a difference between maximal and minimal myocardial ATs. The standard deviation of reconstructed myocardial AT (SDATm) was also computed.

### Study protocol

The BSM data were recorded in sinus rhythm (pacing off) and during six distinct pacing configurations in each patient, including atrial pacing, sequential left ventricular (LV) or right ventricular (RV), and biventricular pacing (BVP). All pacing modes were set at the same rate 10 b.p.m. above the sinus rhythm rate. Sequential LV or RV pacing modes were programmed with an AV delay of 120 ms. BVP modes were programmed with AV delay 120 ms and VV delay 0 ms, with AV delay programmed 20 ms shorter than the spontaneous PQ interval and VV delay 0 ms, and with AV delay programmed 20 ms shorter than the spontaneous PQ interval and VV delay −40 ms. The BSM data were also obtained at the baseline programmed setting before and after applying the experimental pacing protocol to exclude the mutual influence of pacing modes. The BSM data were recorded within 30 s for each programmed pacing mode, starting 15 s after initiation of pacing.

### Statistics

Data are expressed as mean and standard deviation or as the median and interquartile range (IQR) if asymmetric distribution. Statistical analysis was performed with the SPSS package (IBM SPSS Statistics 23). A paired Student’s *t*-test and repeated measures analysis of variance with *post hoc* analysis by a Bonferroni adjustment were applied for paired and multiple comparisons, respectively. Reliability analysis with intraclass correlation coefficient (ICC, two-way mixed model, absolute agreement type) was performed to assess the similarity of electrophysiological parameters obtained from 12-lead ECG and 96-lead BSM. Pearson’s pairwise test was applied to find a relation between body surface parameters of dyssynchrony with myocardial depolarization heterogeneity. The differences were considered significant at *P* < 0.05.

## Results

### Patient data

**Figure 2:**
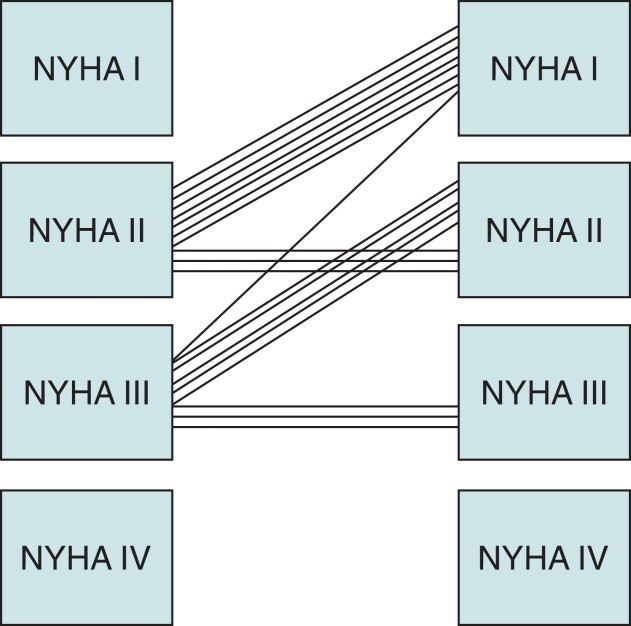
Individual changes in NYHA class after 6 months of CRT. NYHA, New York Heart Association.

The clinical characteristics of 19 patients (58% male) studied are summarized in *Table 1*. The patients were 60 ± 11 years old with a QRS duration of 167 ± 16 ms and median LVEF 25 (IQR 10) prior to CRT implanting. All were in sinus rhythm with true left bundle branch block (LBBB) pattern on ECG. The patients had BVP set empirically after implant with an AV delay −20 ms of the intrinsic PQ interval with a VV delay of 0 ms. At the time of the study, all patients had a CRT device implanted minimum 6 months (median period after the implant was 13 months with IQR 7–66). Twelve patients improved their functional status by NYHA Class I, and the rest remained unchanged (2.47 ± 0.8 vs. 1.74 ± 0.7, *P* < 0.00002, *Figure 2*). Left ventricular ejection fraction (LVEF) improved in all subjects—in 12 by 10%, in 5 by 5%, and in 2 by 20% or more %.

**Table 1:** Clinical characteristics of patients

Characteristic	*n* = 19
Age, years	60 ± 11
Male gender	11 (58%)
NYHA Class II, %	10 (53%)
NYHA Class III, %	9 (47%)
QRS duration, ms	167 ± 16
Systolic pressure, mmHg	123 ± 17
Diastolic pressure, mmHg	76 ± 10
LVEF, %	25 (IQR 10)
Diabetes	3 (16%)
Hypertension	9 (47%)
Beta-blockers	17 (90%)
ACEI/ARB/ARNI	19 (100%)

Values are presented as mean ± standard deviation or median and interquartile range (IQR) if there is asymmetric distribution. Categorical variables are presented as frequencies and percentages.

ACEI/ARB/ARNI, angiotensin-converting enzyme inhibitor or angiotensin II receptor blocker, or angiotensin receptor-neprilysin inhibitor; EF, ejection fraction; LBBB, left bundle branch block; LV, left ventricular; NYHA, New York Heart Association; RBBB, right bundle branch block.

### Standard deviation of activation time as a measure of electrical dyssynchrony

**Figure 3:**
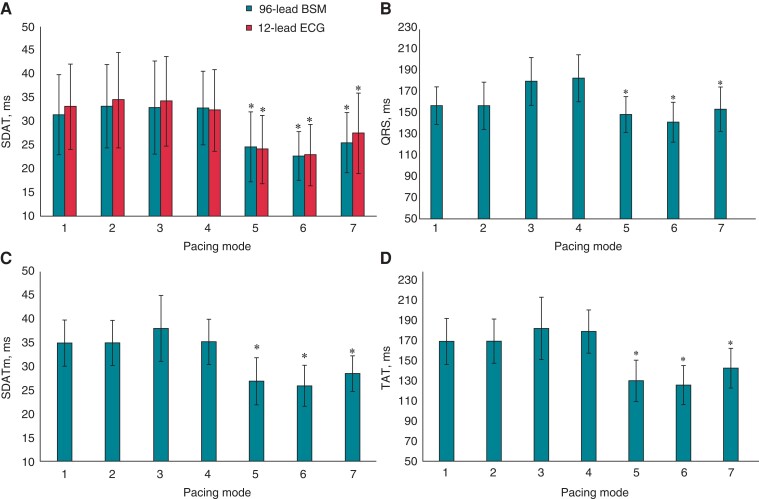
Measures of electrical dyssynchrony in different pacing modes. (*A*) Standard deviation of activation time measured from 96-lead mapping system (96-lead SDAT) and from 12-lead ECG; (*B*) mean QRS duration calculated from standard limb leads; (*C*) SDATm; and (*D*) TAT determined from reconstructed myocardial activation times. Pacing modes: 1, Sinus rhythm; 2, atrial pacing; 3, sequential LV pacing (AV delay 120 ms); 4, sequential RV pacing (AV delay 120 ms); 5, sequential biventricular pacing (AV delay 120 ms, VV delay 0 ms); 6, sequential biventricular pacing (AV delay −20 ms intrinsic PQ, VV delay 0 ms); 7, sequential biventricular pacing (AV delay −20 ms intrinsic PQ, VV delay LV −40 ms over RV). *P < 0.01 in comparison with Pacing Mode 1. SDAT, standard deviation of activation time; SDATm, standard deviation of reconstructed myocardial activation time; TAT, total activation time.

During intrinsic sinus rhythm, atrial pacing, and both sequential RV and LV pacings, the averaged SDAT was predictably higher than the averaged SDAT value obtained in BVP modes. For BSM, it was 32.5 ± 5 vs. 24.2 ± 5 ms (*P* < 0.0001), and for 12-lead ECG, it was 33.5 ± 7 vs. 24.8 ± 6 ms (*P* = 0.0002). The lowest value of SDAT was found for two BVP configurations: sequential BVP (AV delay 120 ms, VV delay 0 ms) and sequential BVP (AV delay −20 ms of intrinsic PQ, VV delay 0 ms; *Figure 3A*). The values of SDAT obtained from 96-lead BSM did not differ from SDAT derived from 12-lead ECG in all pacing modes.

To validate the SDAT as a measure of electrical dyssynchrony, TAT and SDATm reconstructed from ECGi were compared. The changes of TAT and SDATm during different pacing configurations were similar to body surface-derived SDAT (*Figure 3*). In intrinsic rhythm, the LV activation was delayed due to LBBB and this was reflected by a high dispersion of AT values. Similarly, AT dispersion persisted to the similar extent during the RV or LV sequential pacing. However, myocardial dispersion of depolarization was low during different protocols of BVP, especially during sequential BVP with AV delay −20 ms of intrinsic PQ and VV delay 0 ms (*Figure 3C* and *D*). Statistically significant correlation was found between 96-lead SDAT and both TAT (*r* = 0.539, *P* < 0.0001) and SDATm (*r* = 0.510, *P* < 0.0001). Standard deviation of activation time derived from 12-lead ECG also demonstrated significant correlation with TAT (*r* = 0.555, *P* < 0.0001) and SDATm (*r* = 0.513, *P* < 0.0001).

The QRS duration measured from standard limb leads significantly decreased during BVP in comparison with sinus rhythm, atrial pacing, or sequential RV or LV pacing (*Figure 3B*). The most pronounced shortening of QRS duration was found for sequential BVP with AV delay set 20 ms shorter than spontaneous PQ interval and VV delay of 0 ms. This set-up corresponds to configuration with the highest degree of fusion with spontaneous intraventricular conduction. For this configuration, the lowest value of SDAT was found. However, the correlation of QRS duration with SDAT derived from 96-lead BSM or 12-lead ECG was of medium level (*r* = 0.500, *P* < 0.0001 and *r* = 0.473, *P* < 0.0001, respectively).

### 96-Lead body surface map vs. 12-lead electrocardiogram

**Figure 4:**
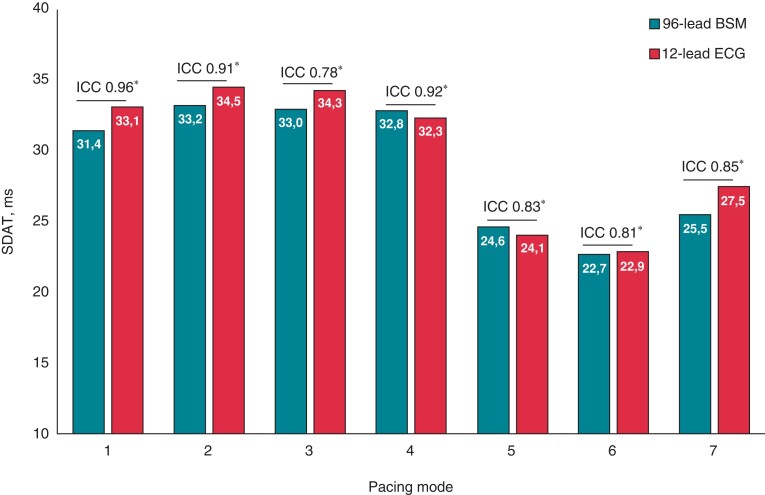
Standard deviation of activation time measured from 96-lead BSM and 12-lead ECG at different pacing modes in CRT patients. Pacing modes: 1, sinus rhythm; 2, atrial pacing; 3, sequential LV pacing (AV delay 120 ms); 4, sequential RV pacing (AV delay 120 ms); 5, sequential biventricular pacing (AV delay 120 ms, VV delay 0 ms); 6, sequential biventricular pacing (AV delay −20 ms intrinsic PQ, VV delay 0 ms); 7, sequential biventricular pacing (AV delay −20 ms intrinsic PQ, VV delay LV −40 ms over RV). BSM, body surface mapping; ECG, electrocardiogram; ICC, intraclass correlation coefficient; SDAT, standard deviation of activation time. *P < 0.001.

Standard deviation of activation time values obtained from 96-lead BSM were close to those derived from 12-lead ECG in sinus rhythm. Similarly, analogous conformity between two measurement methods was demonstrated for SDAT values during different pacing modes, including atrial pacing, sequential LV or RV pacing with AV delay 120 ms, and BIV pacing modes (*Figure 4*).

Reliability analysis with intraclass correlation coefficient (ICC) was performed to test how strongly the values of SDAT quantified by 96-lead mirror the values SDAT from 12-lead ECG. ICC range between 0.78 [95% confidence interval (CI) 0.42–0.92, *P* = 0.002] and 0.96 (95% CI 0.90–0.99, *P* < 0.0001; *Table 2*) for different pacing modes documented that SDAT obtained from BSM was significantly consistent with SDAT calculated from 12-lead ECG. The excellent similarity between mean QRS duration measured from 12-lead ECG and 96-lead BSM was revealed for all pacing configurations with ICC 0.98 (95% CI 0.93–0.99, *P* < 0.0001).

**Table 2:** Similarity of SDAT (ms) measured from 96-lead BSM and 12-lead ECG

Pacing mode	SDAT, ms		ICC	95% CI	*P*-value
	BSM	12-Lead ECG			
Sinus rhythm	31.4 ± 8	33.1 ± 9	0.96	0.90–0.99	<0.0001
A-pacing	33.2 ± 9	34.5 ± 10	0.91	0.77–0.97	<0.0001
Sequential LV pacing	33.0 ± 10	34.3 ± 9	0.78	0.42–0.92	0.002
Sequential RV pacing	32.8 ± 8	32.3 ± 9	0.92	0.78–0.97	<0.0001
Sequential BVP	24.6 ± 7	24.1 ± 7	0.83	0.53–0.94	<0.0001
AV delay 120 ms, VV simultaneous					
Sequential BVP	22.7 ± 5	22.9 ± 6	0.81	0.47–0.93	0.001
AV delay –20 ms intrinsic PQ, VV simultaneous					
Sequential BVP	25.5 ± 6	27.5 ± 9	0.85	0.60–0.94	<0.0001
AV delay –20 ms intrinsic PQ, VV delay –40 ms over RV					

Data are presented as mean ± standard deviation.

BVP, biventricular pacing; CI, confidence interval; ICC, intraclass correlation coefficient.

## Discussion

The results of this study can be summarized as follows: (i) a significant correlation was found between reconstructed myocardial metrics of depolarization heterogeneity (TAT, SDATm) and SDAT derived both from 96- or 12-lead ECG in sinus rhythm and during different pacing configurations, thus validating the SDAT parameter as a measure of dyssynchrony, (ii) compared with spontaneous activation and RV or LV pacing, BVP with the two specific pacing regimes resulted in the lowest value of SDAT, implying that this strategy provides the most efficient electrical resynchronization, (iii) although the QRS duration was shortest for these two BVP configurations, medium-sized correlation was found between the QRS duration and SDAT, supporting the use of SDAT for assessment of electrical dyssynchrony, and most importantly, and (iv) SDAT values obtained from 96-lead BSM were similar to SDAT calculated from the 12-lead ECG, demonstrating strong agreement between both ECG recording methods. If confirmed, it may simplify non-invasive assessment of electrical dyssynchrony.

### Standard deviation of activation time as a measure of electrical dyssynchrony

Earlier studies have documented that SDAT derived from BSM has the potential to predict clinical response to CRT. More recently, SDAT obtained from limited body surface multichannel ECGs (ECG Belt) has been proposed as the equally reliable parameter,^12^ which was validated through comparison with ECGi method of reconstructed ATs on the surface of the heart.^7^ This parameter could be used for guidance of the LV lead placement^5^ and/or for optimization of CRT programming.^6^ It was also found useful in patients less likely to be improved by.^13^

In our study, we performed similar comparison of ECGi-derived parameters of electrical dyssynchrony such as TAT or SDATm with SDAT from 96-lead BSM and also from 12-lead ECG. Compared with the previous study, which found a strong correlation between values of SDAT calculated from reconstructed epicardial potentials (ECGi) and body surface potentials,^7^ our findings indicated lower strength of the relationship between SDAT values derived from BSM and reconstructed myocardial ATs. These differences may relay to different inverse solution approaches. An alternative explanation for different results may be related to another difference in methodology. While the above study used only reconstructed epicardial potentials,^7^ our technique also employed endo- and epicardial myocardial ATs, including septal. It is also important to emphasize that SDAT as a metric of electrical dyssynchrony has not been validated by direct measurements of myocardial ATs on the surface of the heart. On the other hand, other studies demonstrated the correlation of SDAT with acute haemodynamic response to pacing.^5^ Also recent comprehensive review of the literature identified SDAT as a most promising non-invasive parameter for assessment of electrical dyssynchrony.^4^

Importantly, our study compared SDAT values in individual pacing setups in a cohort of patients with non-ischaemic cardiomyopathy and found the lowest values for two of them, both BVP regimes, supporting the previous studies. Interestingly, these two BVP setups did use 0 VV delay and AV delay fixed at 120 or 20 ms shorter than the spontaneous PQ interval. Such settings should allow in patients with preserved AV conduction relatively high degree of fusion of paced wavefronts with a spontaneous activation via the conduction system. Such presumption appears to be confirmed by parallel shortening of the mean QRS duration in those two pacing configurations and by some correlation between SDAT values and the QRS duration. Interestingly, BVP configuration with advanced LV wavefront (i.e. VV delay −40 ms) resulted in higher SDAT values. This configuration is also characterized by a broader QRS complex.

Based on our previous experience, we use a pragmatic approach for the setting of AV delay in CRT patients without AV block in our practice. We use AV delay 20 ms shorter than spontaneous AV interval or, alternatively, fixed AV delay of 120 ms. Interestingly, this study confirmed that these configurations provided the shortest SDAT. Many patients with this setting had improvement in functional status and all had some improvement in LVEF. Based on these findings, further studies of SDAT as a metric of electrical dyssynchrony have to be performed, evaluating this parameter in different categories of patients, including responders and non-responders to CRT.

### Body surface map vs. 12-lead electrocardiogram

The most important finding of our study is that SDAT can be calculated from the 12-lead ECG instead of 96-lead BSM with the same diagnostic yield. The 12-lead ECG-derived SDAT values were in good agreement with BSM-derived SDAT values for all pacing configurations and for the intrinsic ventricular activation, i.e. sinus rhythm or atrial pacing (*Figure 4*). Further prospective studies of SDAT derived from 12-lead ECG as a metric of electrical dyssynchrony need to be conducted to confirm our preliminary results. Especially subjects with ischaemic cardiomyopathy have to be studied.

Interestingly, the advantage of SDAT obtained from the BSM system over the QRS duration was demonstrated in an earlier study using native rhythm and baseline CRT settings.^6^ The authors did not find a correlation between these two metrics of electrical synchrony. When compared with QRS duration measured in 12-lead ECG, SDAT from BSM demonstrated better predictive ability for LV remodelling in response to CRT.^12,14^ These observations indicating SDAT as a more sensitive metric of electrical dyssynchrony than QRS duration were not supported by our results. Changes in QRS duration paralleled SDAT changes during different pacing modes, and there was a reasonable correlation between SDAT and QRS. This controversy may reflect the differences in the studied cohorts. Our study specifically included non-ischaemic patients with LBBB, who have more homogeneous intramyocardial conduction compared with ischaemic patients and a higher probability of being responders.

### Limitations

The study was performed on a relatively small population of patients with non-ischaemic cardiomyopathy and true LBBB. This limits the generalization of the results to patients with ischaemic cardiomyopathy or patients with other patterns of intraventricular conduction abnormalities. On the other hand, we selected non-ischaemic cardiomyopathy as a model for such studies focused on proof of concept. Notably, the measured parameters in the studied population had normal distribution, and data analysis demonstrated statistically significant results. Another limitation of this study relates to 12-lead ECG electrode positions, since precordial unipolar leads (V1–V6) were selected from the array of 96-lead BSM, and thus, the position of these electrodes slightly varied from the standard position of these leads. Finally, we do not have measurements that would allow a comparison of SDAT values in individual pacing configurations with their haemodynamic benefit.

## Conclusions

Using BSM for the non-invasive assessment of electrical dyssynchrony, we found the lowest SDAT values for the two BVP configurations, suggesting the most efficient electrical resynchronization. In addition, a significant correlation was found between simulated myocardial metrics of depolarization heterogeneity obtained from ECGi and SDAT derived from BSM, supporting the usefulness of SDAT. Most importantly, SDAT calculated from the 12-lead ECG provided similar results as BSM.

## Data Availability

Due to privacy and ethical concerns, the data cannot be made available to the public.
